# Documentation of social determinants of health for patients with type 2 diabetes in Epic Cosmos

**DOI:** 10.1093/jamiaopen/ooaf095

**Published:** 2025-09-04

**Authors:** Polina V Kukhareva, Matthew J O’Brien, Daniel C Malone, Kensaku Kawamoto, Ramkiran Gouripeddi, Deepika Reddy, Mingyuan Zhang, Vikrant G Deshmukh, David Danks, Julio C Facelli

**Affiliations:** Department of Biomedical Informatics, University of Utah, Salt Lake City, UT 84108, United States; Department of General Internal Medicine, Northwestern University, Chicago, IL 60611, United States; Department of Pharmacotherapy, University of Utah, Salt Lake City, UT 84108, United States; Department of Biomedical Informatics, University of Utah, Salt Lake City, UT 84108, United States; Department of Biomedical Informatics, University of Utah, Salt Lake City, UT 84108, United States; Diabetes and Endocrinology Center, University of Utah, Salt Lake City, UT 84108, United States; Department of Population Health Sciences, University of Utah, Salt Lake City, UT 84102, United States; Department of Biomedical Informatics, University of Utah, Salt Lake City, UT 84108, United States; Department of Computer Science and Engineering, University of California, San Diego, CA 92093, United States; Department of Biomedical Informatics, University of Utah, Salt Lake City, UT 84108, United States

**Keywords:** social determinants of health, electronic health records, type 2 diabetes

## Abstract

**Objectives:**

Type 2 diabetes (T2D) is a growing public health burden with persistent racial and ethnic disparities. . This study assessed the completeness of social determinants of health (SdoH) data for patients with T2D in Epic Cosmos, a nationwide, cross-institutional electronic health recors (EHR) database.

**Materials and Methods:**

The study included adults with T2D (ICD-10: E11.*) with encounters between 2022 and 2024. We analyzed 11 individual-level SDoH data elements across 5 domains—financial strain, food insecurity, housing instability, intimate partner violence, and transportation needs—and 4 components of the Social Vulnerability Index (SVI), representing neighborhood-level SDoH. Data completeness for each data element (ie, the proportion of individuals with non-missing values) was evaluated using generalized linear models, adjusting for source healthcare organization, sex, and age.

**Results:**

Among 12 031 927 individuals with T2D, adjusted completeness for individual-level SDoH data elements ranged from 11.2% to 31.5%, varying by data element and racial/ethnic group. American Indian or Alaska Native, Asian, Hispanic, and Native Hawaiian or Other Pacific Islander individuals had lower completeness for all individual-level SDoH compared to White individuals. In contrast, SVI data elements were available for nearly all patients since they are derived from patient addresses routinely collected in EHRs.

**Discussion:**

While SVI data elements were widely available, individual-level SDoH data elements had significant missingness, limiting their usability for secondary analyses. Racial/ethnic disparities in SDoH completeness further complicate their use.

**Conclusion:**

Standardized, equitable SDoH collection is critical to close documentation gaps, reduce disparities, and enable accurate, bias-resistant analyses in T2D care.

Highlights

1. This study examined social determinants of health data for adults with type 2 diabetes in a cross-institutional electronic health record database to support epidemiological studies and AI model development for type 2 diabetes management.
2. Neighborhood-level social determinants of health data were highly complete, while individual-level social determinants of health data were mostly missing.
3. Disparities in social determinants of health data completeness by race/ethnicity underscore the need for standardized social determinants of health documentation.

## Background

With near-universal adoption and growing data interoperability, electronic health records (EHRs) have become a crucial resource for secondary analyses, including epidemiological studies, health services research, and artificial intelligence (AI) model development.^1^ In recent years, several national real-world de-identified databases that incorporate EHR data have emerged, including Epic Cosmos,^2^ TriNetX,^3^ All of Us,^4^ National COVID Cohort Collaborative (N3C),^5^ Patient-Centered Outcomes Research Network (PCORnet),^6^ and Flatiron Health.^7^ Some national EHR databases are designed either by scope or policy for specific research areas, such as oncology (Flatiron Health), and COVID-19 (N3C). Others, like Epic Cosmos, All of Us, and TriNetX, do not have a specific disease focus. Among them, Epic Cosmos is one of the largest and most detailed EHR databases in the United States.

Social determinants of health (SDoH) are defined as conditions in which people are born, grow, live, work, and age.^8^ World Health Organization reports that SDoH account for between 30% and 55% of health outcomes.^9^ The ability to capture structured SDoH data in EHRs is critical for identifying at-risk populations, addressing health inequities, and improving clinical decision support. Given the increasing recognition of SDoH as key determinants of health outcomes, there is growing interest in using EHRs to systematically capture SDoH data.

Completeness of individual-level SDoH data is particularly important for patients with type 2 diabetes (T2D),^10–12^ a chronic metabolic condition that affected 38.4 million US adults in 2021 and is projected to affect 60.6 million by 2060.^13^^,^^14^ SDoH play a critical role in T2D outcomes, influencing disease onset, management, and disparities in complications and mortality. For example, effective T2D management relies heavily on lifestyle modifications, including dietary changes and regular physical activity. Therefore, food insecurity and financial strain can directly limit a person’s ability to adopt these changes, negatively impacting glycemic control and overall disease management. Furthermore, T2D is one of the most common, well-characterized, and guideline-driven chronic diseases, making it an ideal case study for evaluating SDoH data completeness and its implications for health equity and AI-driven healthcare models. Its high prevalence, strong link to SDoH, and need for frequent healthcare interactions position it as a representative model for assessing SDoH documentation in EHRs, with findings that may be generalizable to other chronic conditions.

Federal healthcare agencies and payers are moving toward standardized SDoH documentation. The Centers for Medicare & Medicaid Services (CMS) are now requiring the collection of “Big 5” SDoH domains—financial strain, food insecurity, housing instability, intimate partner violence (IPV), and transportation needs to improve healthcare equity.^15^ Many health systems are proactively integrating SDoH screening into routine clinical practice,^16^ and Epic Systems, the largest EHR vendor in the United States, is prioritizing SDoH data collection.^17^ Additionally, data interoperability standards are emerging to facilitate the consistent exchange of SDoH data across healthcare institutions.^18^^,^^19^

Despite these efforts, early reports indicate substantial gaps in SDoH completeness in EHR data, raising concerns about whether these data are sufficiently available for secondary research and quality improvement initiatives.^18^^,^^20–23^ However, no comprehensive studies have systematically evaluated SDoH completeness stratified by race/ethnicity in a large, multi-institutional EHR database for individuals with T2D.

This study aims to characterize SDoH data completeness in Epic Cosmos,^24^ focusing on the 5 individual-level SDoH domains and Social Vulnerability Index (SVI) in patients with T2D. Given that T2D disproportionately affects racial and ethnic minority groups,^25^ and that SDoH differ by race/ethnicity, we stratified our analysis by race/ethnicity. By identifying gaps in SDoH capture, this research informs efforts to improve standardized documentation, equitable healthcare delivery and planning of secondary analyses using EHR data.

## Methods

### Study design

The study design is a retrospective cross-sectional study using de-identified EHR data from Epic Cosmos. The REporting of studies Conducted using Observational Routinely-collected health Data (RECORD) guideline was used for reporting.^26^

### Ethical aspects

The University of Utah Institutional Review Board (IRB) reviewed the study proposal and determined that it does not meet the definition of Human Subjects Research, as all data undergo systematic de-identification prior to submission to Epic Cosmos. Consequently, no further IRB review was required.

### Data sources

All variables were derived from structured fields within Epic Cosmos, a dataset created in collaboration with a community of Epic health systems representing more than 295 million patient records from over 1633 hospitals and 37 900 clinics from all 50 states, D.C., Lebanon, and Saudi Arabia. The current count values for patients, hospitals, and clinics are available on cosmos.epic.com (as of January 2025).^2^ Data extraction was completed on January 21, 2025. No linkage to other data sources (eg, insurance claims or patient surveys) was performed.

### Setting

These data came from 208 source organizations in the United States, including healthcare systems, hospitals, and clinics.

### Participants

Inclusion criteria were as follows:

- Adults aged 18 years or older.
- A documented diagnosis of type 2 diabetes (ICD-10 code E11.*) between January 1, 2020, and December 31, 2024.
- At least one clinical encounter (office visit, telemedicine appointment, surgical procedure, or laboratory test) in the United States between January 1, 2022, and December 31, 2024.
- Records contributed by health systems participating in Epic Cosmos that had submitted complete and continuous data starting on or before December 31, 2020.

We excluded patients who identified with more than one racial category, as this heterogeneous group would require a more detailed analysis than was feasible within the scope of this study.

### Variables

**Figure 1.**
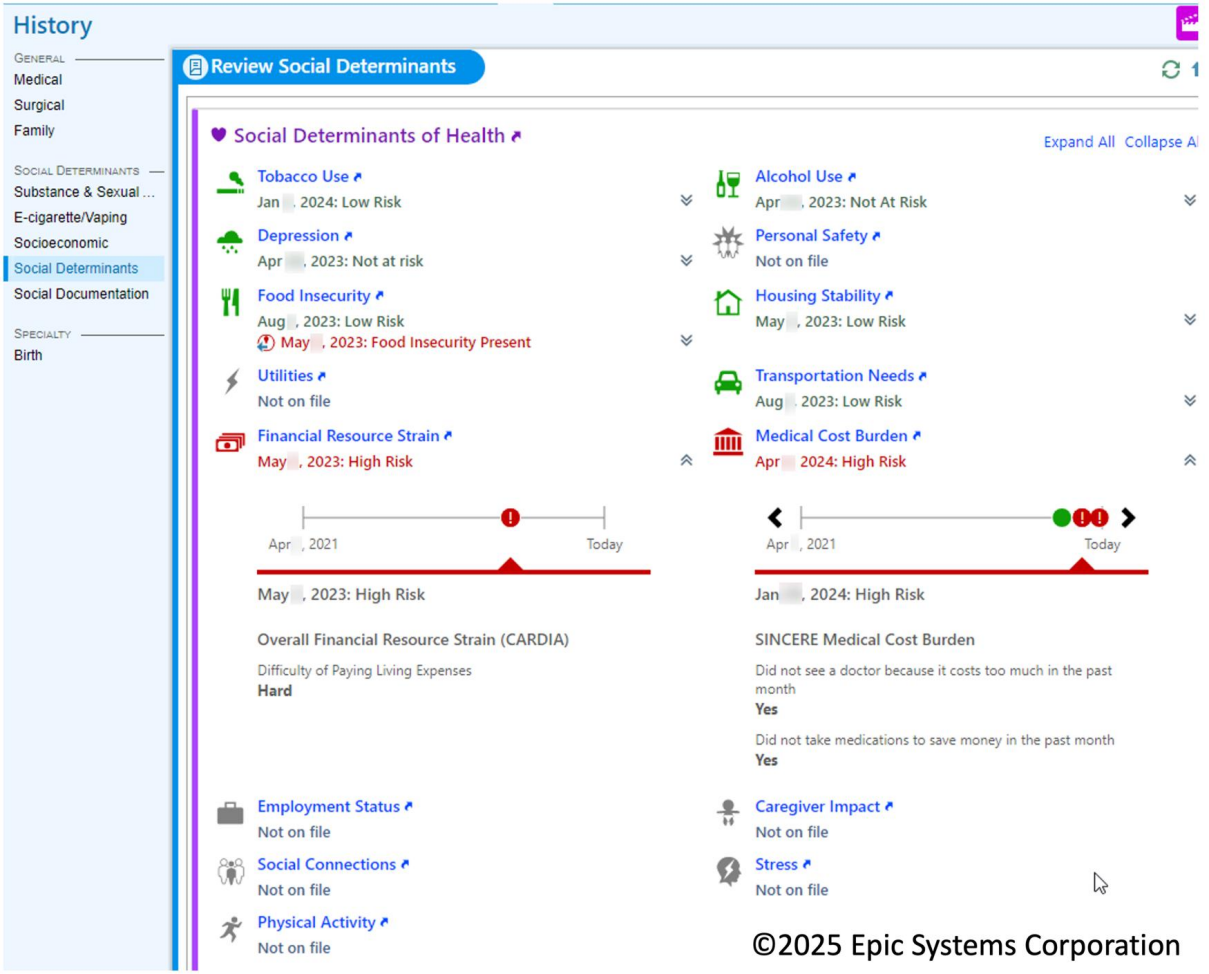
SDoH data captured and displayed in the EHR.

We examined both individual-level and neighborhood-level SDoH data elements. Individual-level SDoH included only the most recent answers for a patient from an organization. CMS Big 5 SDoH variables are documented in the EHR by clinical providers and medical support staff using integrated SDoH displays (Figure 1). Some of these questions come from Accountable Health Communities (AHC) Health-Related Social Needs (HRSN) Screening Tool and the Protocol for Responding to and Assessing Patients’ Assets, Risks, and Experiences (PRAPARE) Tool. During data extraction, transformation, standardization, de-identification, and aggregation across participating healthcare organizations, certain variables may be modified or omitted. We have prioritized evaluation of CMS-designated “Big 5” domains (financial strain, food insecurity, housing instability, intimate partner violence, and transportation barriers). Big 5 domains were represented by 11 data elements: financial resource strain, food scarcity, food worry, housing (homelessness, mortgage), IPV (emotional, fear, physical abuse, sexual abuse), medical transportation needs, and non-medical transportation needs (Table 1). For individuals who had multiple SDoH values recorded, we used the last value collected prior to the data extraction date.

**Table 1.** Epic Cosmos Big 5 SDoH variables.

Domain	Data element	Definition	Values
**Individual-level SDoH**			
Financial strain	Financial strain	The patient’s response to the question: “How hard is it for you to pay for the very basics like food, housing, medical care, and heating?”	Not hard at all, Not very hard, Somewhat hard, Hard, Very hard
Food insecurity	Scarcity	The patient’s response to the statement: “Within the past 12 months, the food you bought just didn’t last and you didn’t have money to get more.”	Never true, Sometimes true, Often true
	Worry	The patient’s response to the statement: “Within the past 12 months, you worried that your food would run out before you got the money to buy more.”	Never true, Sometimes true, Often true
Housing instability	Homelessness	The patient’s response to the question: “In the last 12 months, was there a time when you did not have a steady place to sleep or slept in a shelter (including now)?”	No, Yes
	Mortgage	The patient’s response to the question: “In the last 12 months, was there a time when you were not able to pay the mortgage or rent on time?”	No, Yes
IPV	Emotional	The patient’s response to the question: “Within the last year, have you been humiliated or emotionally abused in other ways by your partner or ex-partner?”	No, Yes
	Fear	The patient’s response to the question: “Within the last year have you been afraid of your partner or ex-partner?”	No, Yes
	Physical abuse	The patient’s response to the question: “Within the last year, have you been kicked, hit, slapped, or otherwise physically hurt by your partner or ex-partner?”	No, Yes
	Sexual abuse	The patient’s response to the question: “Within the last year, have you been raped or forced to have any kind of sexual activity by your partner or ex-partner?”	No, Yes
Transportation needs	Medical	The patient’s response to the question: “Has the lack of transportation kept you from medical appointments or from getting medications?”	No, Yes
	Non-medical	The patient’s response to the question: “Has the lack of transportation kept you from meetings, work, or from getting things needed for daily living?”	No, Yes
**Neighborhood-level SDoH**			
SVI	Socioeconomic status	Socioeconomic status reflects economic disadvantage and includes poverty rate, unemployment, per capita income, and educational attainment.	[0, 1]
	Household characteristics	Household characteristics capture vulnerabilities related to age, disability, and family structure, including the proportion of elderly individuals, children, single-parent households, and individuals with disabilities.	[0, 1]
	Racial and ethnic minority status	Racial and ethnic minority status measures the proportion of racial/ethnic minority populations and individuals with limited English proficiency, recognizing disparities in access to healthcare and resources.	[0, 1]
	Housing and transportation	Housing and transportation assesses residential stability and mobility limitations, incorporating the proportion of multi-unit housing, mobile homes, overcrowding, households without a vehicle, and individuals living in group quarters.	[0, 1]

Abbreviations: IPV, intimate partner violence; SVI, social vulnerability index.

Unlike individual-level SDoH variables, which reflect a person’s specific circumstances, neighborhood-level SDoH are derived from an individual’s residential location and may be less precise for assessing personal social risk. In this study, we analyzed 4 components of the Social Vulnerability Index (SVI)—socioeconomic status, racial and ethnic minority status, household characteristics, and housing and transportation (Table 1).^27^ We used the 2018 SVI at the ZIP code level as a measure of neighborhood-level SDoH. The SVI, developed by the Centers for Disease Control and Prevention (CDC), helps public health officials and policymakers identify communities that may require additional support during emergencies such as natural disasters, disease outbreaks, or environmental hazards. It is based on 16 US Census variables, which collectively assess a community’s capacity to prepare for, respond to, and recover from public health crises. SVI scores range from 0 (least vulnerable) to 1 (most vulnerable), with higher scores indicating greater social vulnerability. In our analysis, we defined high vulnerability as falling into the most vulnerable quartile (SVI values from 0.75 to 1) for each component.

Racial and ethnic classifications followed the 2024 Office of Management and Budget (OMB) standards, which recognize 7 categories: American Indian or Alaska Native (AI/AN), Asian, Black or African American (Black), Hispanic or Latino (Hispanic), Middle Eastern or North African (MENA), Native Hawaiian or Pacific Islander (NH/PI), and White.^28^ Data on MENA identity were not available in the dataset, despite its recent inclusion as a distinct category. Hispanic individuals of any race were aggregated into the Hispanic category, while all other categories were treated as Non-Hispanic (eg, Non-Hispanic White, Non-Hispanic Black).

### Bias

This study may be subject to several forms of bias inherent to observational research using EHR data. Documentation bias is likely, as SDoH information may be selectively recorded based on provider discretion, patient disclosure, or available workflows. Additionally, differential completeness by race and ethnicity introduces potential for measurement bias, which could impact both prevalence estimates and downstream analyses.

### Data access and cleaning methods

All data were accessed within the secure Epic Cosmos portal by authorized researchers at the University of Utah. Data underwent automated standardization and de-identification by Epic Cosmos team prior to analysis. Structured SDoH fields were retained as-is, and no additional cleaning or transformation was conducted beyond variable selection and consistency checks.

### Statistical analysis

We assessed 3 types of outcomes: completeness, prevalence of social needs, and prevalence of social vulnerability. Completeness was defined as the proportion of patients with non-missing values for each SDoH variable. Social need prevalence was defined as the proportion of non-missing responses indicating unmet need (eg, answers other than “No,” “Never true,” or “Not hard at all”). Social vulnerability was defined as having an SVI domain score in the highest quartile.

Descriptive characteristics were summarized using counts (%) for sex. Mean (standard deviation) were used for age and SVI. Results with less than 10 observations are reported as <10 to prevent re-identification. Number of patients with documented social needs was reported to inform sample size estimations for researchers planning to conduct analyses based on identified sub-population reporting unmet needs.

To estimate the proportion of individuals with documented SDoH variables, we used a generalized linear model (GLM) with a log link function and a binomial distribution, employing generalized estimating equations (GEE) to account for the hierarchical structure of EHR data. GEE was chosen to adjust for clustering within source healthcare organizations using an exchangeable correlation structure, providing population-averaged estimates. The model adjusted for age and sex and was based on a random sample of 5000 patients per racial/ethnic group (N = 30 000). Results are reported as estimated marginal means and odds ratios (ORs). To enhance interpretability and facilitate comparisons across racial/ethnic groups, ORs are presented with White individuals as the reference category. The type I error rate was set at 0.05.

## Results

**Figure 2.**
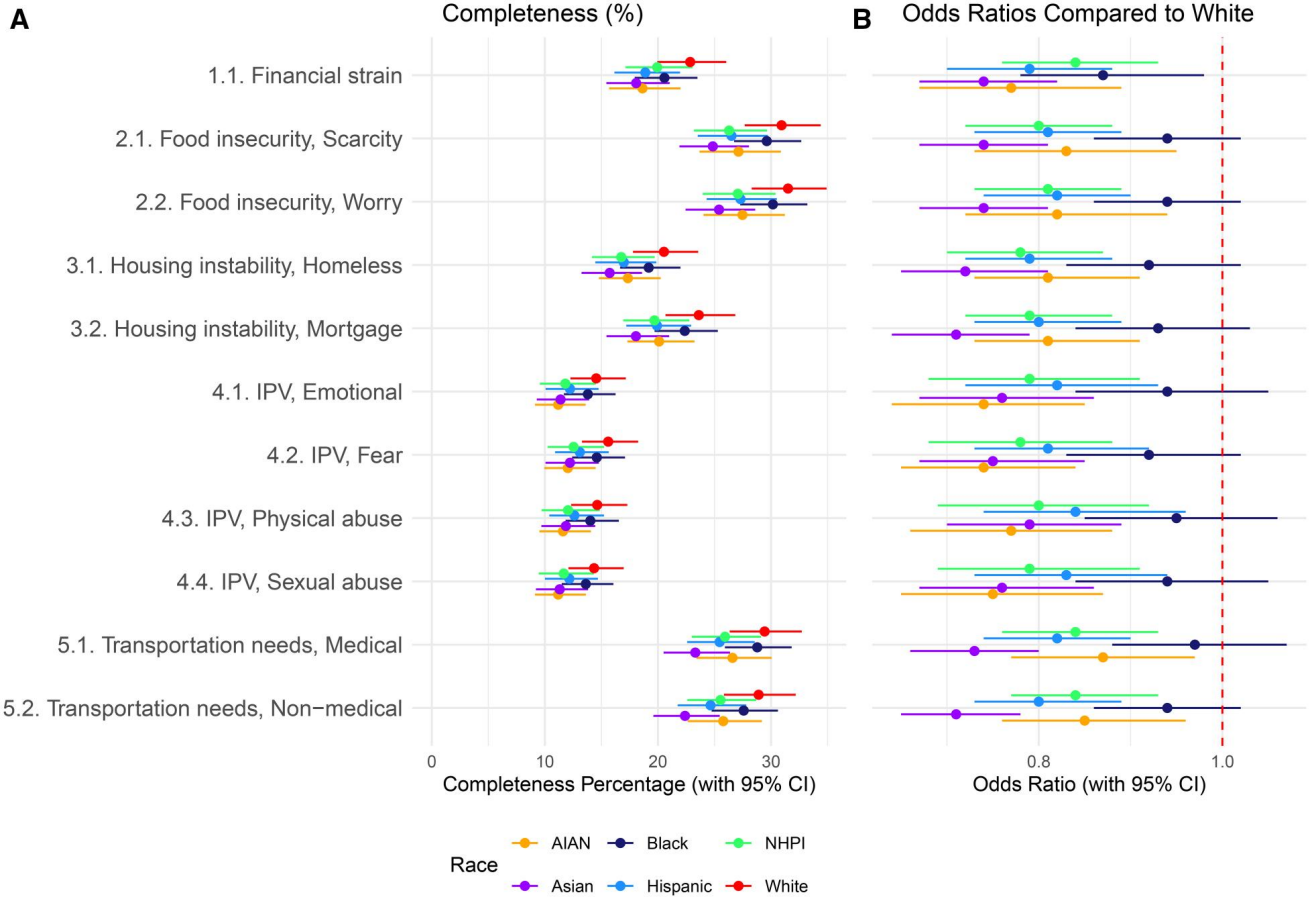
Completeness of SDoH variables among patients with T2D by race/ethnicity. (A) Percentage of complete records. (B) Odds ratios compared to White Individuals. All values were calculated using logistic regression and adjusted for age and sex. Abbreviations: AI/AN, American Indian or Alaskan Native; Black, Black or African American; Hispanic, Hispanic or Latino; NH/PI, Native Hawaiian or Other Pacific Islander.

**Figure 3.**
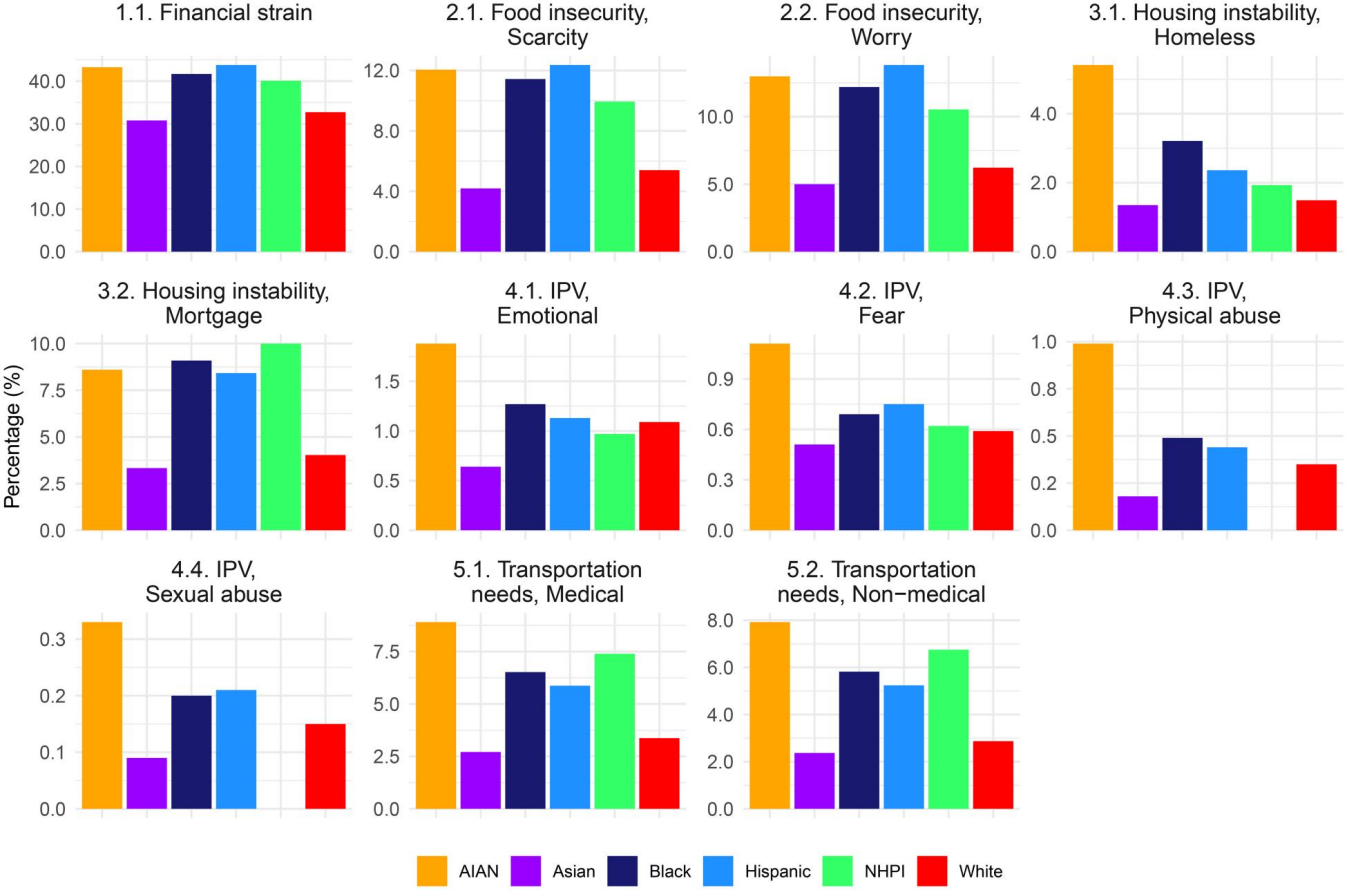
Presence of social needs among patients with T2D by race/ethnicity. Abbreviations: AI/AN, American Indian or Alaskan Native; Black, Black or African American; Hispanic, Hispanic or Latino; NH/PI, Native Hawaiian or Other Pacific Islander.

Table 2 displays the demographic characterization of the 12 031 927 individuals with T2D included in this study. This represents approximately one-third of all individuals in the United States with T2D. The average age was 65.12 (SD: 14.77) years, and 50.1% were female. Completeness of SDoH variables by race/ethnicity is summarized in Figure 2 and Table 3. The remaining study outcomes (prevalence of social needs and prevalence of social vulnerability) are summarized in Figure 3 and Table 4. Below we discuss the findings for each of the Big 5 SDoH domains.

**Table 2.** Patient demographics by race/ethnicity.

Characteristic	White	AI/AN	Asian	Black	Hispanic	NH/PI	Overall
Eligible patients, N	7 635 134	61 775	435 819	2 228 511	1 636 802	33 886	12 031 927
Age, years, mean (SD)	67.63 (14.04)	60.67 (15.01)	64.84 (14.94)	62.2 (15.11)	59.39 (15.36)	59.88 (14.7)	65.34 (14.82)
Legal sex, N (%)							
Female	358 8338 (47)	32 953 (53.34)	220 177 (50.52)	1 278 827 (57.38)	867 546 (53)	17 950 (52.97)	6 005 791 (49.92)
Male	4 046 196 (52.99)	28 818 (46.65)	215 583 (49.47)	949 503 (42.61)	769 071 (46.99)	15 933 (47.02)	6 025 104 (50.08)
Other	26 (0)	<10 (Masked)	<10 (Masked)	<10 (Masked)	15 (0)	<10 (Masked)	45 (0)
Unknown	574 (0.01)	<10 (Masked)	59 (0.01)	177 (0.01)	170 (0.01)	<10 (Masked)	987 (0.01)

Abbreviations: AI/AN, American Indian or Alaskan Native; Black, Black or African American; Hispanic, Hispanic or Latino; NH/PI, Native Hawaiian or Other Pacific Islander.

**Table 3.** Completeness of SDoH variables among patients with T2D by race/ethnicity.

Domain	Element	White	AI/AN	Asian	Black	Hispanic	NH/PI
**Individual-level SDoH**							
Financial strain	Financial strain	22.85 (19.94-26.04)	18.63 (15.68-21.99), OR: 0.77 (0.67-0.89)	18.07 (15.44-21.04), OR: 0.74 (0.67-0.82)	20.57 (17.94-23.49), OR: 0.87 (0.78-0.98)	18.88 (16.15-21.95), OR: 0.79 (0.7-0.88)	19.93 (17.13-23.05), OR: 0.84 (0.76-0.93)
Food insecurity	Scarcity	30.93 (27.67-34.39)	27.12 (23.67-30.87), OR: 0.83 (0.73-0.95)	24.85 (21.9-28.05), OR: 0.74 (0.67-0.81)	29.62 (26.73-32.67), OR: 0.94 (0.86-1.02)	26.5 (23.52-29.72), OR: 0.81 (0.73-0.89)	26.29 (23.18-29.65), OR: 0.8 (0.72-0.88)
	Worry	31.50 (28.29-34.91)	27.47 (24.02-31.22), OR: 0.82 (0.72-0.94)	25.4 (22.44-28.6), OR: 0.74 (0.67-0.81)	30.16 (27.27-33.21), OR: 0.94 (0.86-1.02)	27.3 (24.31-30.51), OR: 0.82 (0.74-0.9)	27.06 (23.96-30.4), OR: 0.81 (0.73-0.89)
Housing instability	Homeless	20.53 (17.79-23.56)	17.34 (14.78-20.24), OR: 0.81 (0.73-0.91)	15.73 (13.24-18.59), OR: 0.72 (0.65-0.81)	19.18 (16.65-21.99), OR: 0.92 (0.83-1.02)	16.98 (14.46-19.85), OR: 0.79 (0.72-0.88)	16.75 (14.16-19.71), OR: 0.78 (0.7-0.87)
	Mortgage	23.61 (20.67-26.84)	20.1 (17.3-23.23), OR: 0.81 (0.73-0.91)	18.05 (15.45-20.99), OR: 0.71 (0.64-0.79)	22.37 (19.7-25.29), OR: 0.93 (0.84-1.03)	19.91 (17.2-22.93), OR: 0.8 (0.73-0.89)	19.69 (16.93-22.78), OR: 0.79 (0.72-0.88)
IPV	Emotional	14.54 (12.26-17.16)	11.18 (9.13-13.61), OR: 0.74 (0.64-0.85)	11.39 (9.28-13.91), OR: 0.76 (0.67-0.86)	13.8 (11.68-16.25), OR: 0.94 (0.84-1.05)	12.22 (10.07-14.75), OR: 0.82 (0.72-0.93)	11.81 (9.56-14.5), OR: 0.79 (0.68-0.91)
	Fear	15.60 (13.28-18.25)	12.03 (9.94-14.49), OR: 0.74 (0.65-0.84)	12.22 (10.06-14.78), OR: 0.75 (0.67-0.85)	14.59 (12.41-17.09), OR: 0.92 (0.83-1.02)	13.09 (10.9-15.65), OR: 0.81 (0.73-0.92)	12.54 (10.26-15.23), OR: 0.78 (0.68-0.88)
	Physical abuse	14.63 (12.32-17.29)	11.6 (9.52-14.07), OR: 0.77 (0.66-0.88)	11.86 (9.69-14.45), OR: 0.79 (0.7-0.89)	14.02 (11.83-16.54), OR: 0.95 (0.85-1.06)	12.61 (10.39-15.23), OR: 0.84 (0.74-0.96)	12.05 (9.71-14.86), OR: 0.8 (0.69-0.92)
	Sexual abuse	14.35 (12.08-16.97)	11.18 (9.12-13.63), OR: 0.75 (0.65-0.87)	11.31 (9.21-13.82), OR: 0.76 (0.67-0.86)	13.62 (11.49-16.06), OR: 0.94 (0.84-1.05)	12.16 (10.01-14.69), OR: 0.83 (0.73-0.94)	11.67 (9.45-14.33), OR: 0.79 (0.69-0.91)
Transportation needs	Medical	29.43 (26.34-32.72)	26.59 (23.4-30.05), OR: 0.87 (0.77-0.97)	23.3 (20.5-26.37), OR: 0.73 (0.66-0.8)	28.78 (25.92-31.82), OR: 0.97 (0.88-1.07)	25.45 (22.58-28.55), OR: 0.82 (0.74-0.9)	25.93 (22.99-29.11), OR: 0.84 (0.76-0.93)
	Non-medical	28.9 (25.83-32.18)	25.77 (22.63-29.19), OR: 0.85 (0.76-0.96)	22.39 (19.6-25.46), OR: 0.71 (0.65-0.78)	27.58 (24.74-30.61), OR: 0.94 (0.86-1.02)	24.65 (21.74-27.81), OR: 0.8 (0.73-0.89)	25.53 (22.6-28.69), OR: 0.84 (0.77-0.93)
**Neighborhood-level SDoH**							
SVI	Socioeconomic status	99.29 (98.35-99.70)	94.68 (91.55-96.69), OR: 0.13 (0.06-0.27)	99.35 (97.48-99.84), OR: 1.1 (0.56-2.16)	99.11 (97.76-99.65), OR: 0.8 (0.52-1.21)	99.06 (96.87-99.72), OR: 0.75 (0.44-1.27)	99.05 (97.05-99.7), OR: 0.74 (0.37-1.49)
	Household characteristics	99.29 (98.35-99.70)	94.68 (91.55-96.69), OR: 0.13 (0.06-0.27)	99.35 (97.48-99.84), OR: 1.1 (0.56-2.16)	99.11 (97.76-99.65), OR: 0.8 (0.52-1.21)	99.06 (96.87-99.72), OR: 0.75 (0.44-1.27)	99.05 (97.05-99.7), OR: 0.74 (0.37-1.49)
	Racial and ethnic minority status	99.29 (98.35-99.70)	94.68 (91.55-96.69), OR: 0.13 (0.06-0.27)	99.35 (97.48-99.84), OR: 1.1 (0.56-2.16)	99.11 (97.76-99.65), OR: 0.8 (0.52-1.21)	99.06 (96.87-99.72), OR: 0.75 (0.44-1.27)	99.05 (97.05-99.7), OR: 0.74 (0.37-1.49)
	Housing and transportation	99.29 (98.35-99.70)	94.68 (91.55-96.69), OR: 0.13 (0.06-0.27)	99.35 (97.48-99.84), OR: 1.1 (0.56-2.16)	99.11 (97.76-99.65), OR: 0.8 (0.52-1.21)	99.06 (96.87-99.72), OR: 0.75 (0.44-1.27)	99.05 (97.05-99.7), OR: 0.74 (0.37-1.49)

Values for White are expressed as estimated mean with 95% confidence intervals. Values for AI/AN, Asian, Black, Hispanic, and NW/PI are reported as estimated mean with 95% CI, followed by odds ratios (ORs) with 95% confidence intervals. All values were calculated using logistic regression and adjusted for age, sex, and organizational source.

Abbreviations: AI/AN, American Indian or Alaskan Native; Black, Black or African American; Hispanic, Hispanic or Latino; IPV, interpersonal violence; NH/PI, Native Hawaiian or Other Pacific Islander; OR, odds ratio; SDoH, social determinants of health; SVI, social vulnerability index.

**Table 4.** Prevalence of social needs among patients with T2D by race/ethnicity.

Domain	Element	White	AI/AN	Asian	Black	Hispanic	NH/PI
**Individual-level SDoH**							
Financial strain	Financial strain	610 089 (32.72)	5925 (43.27)	19 280 (30.78)	222 892 (41.68)	134 325 (43.78)	1600 (40.09)
Food insecurity	Scarcity	129 502 (5.4)	2053 (12.05)	4028 (4.19)	78 418 (11.43)	47 501 (12.36)	900 (9.94)
	Worry	150 872 (6.22)	2234 (12.98)	4923 (5)	84 368 (12.19)	53 922 (13.82)	982 (10.53)
Housing instability	Homeless	23 842 (1.49)	493 (5.41)	723 (1.35)	14 348 (3.21)	6042 (2.36)	65 (1.93)
	Mortgage	72 841 (4.03)	1106 (8.6)	1984 (3.33)	44 992 (9.09)	25 453 (8.42)	371 (10)
IPV	Emotional	11 144 (1.09)	126 (1.88)	209 (0.64)	3903 (1.27)	1917 (1.13)	26 (0.97)
	Fear	6445 (0.59)	77 (1.11)	177 (0.51)	2215 (0.69)	1338 (0.75)	18 (0.62)
	Physical abuse	3600 (0.35)	78 (0.99)	63 (0.18)	1538 (0.49)	771 (0.44)	<10 (Masked)
	Sexual abuse	1535 (0.15)	22 (0.33)	29 (0.09)	625 (0.2)	361 (0.21)	<10 (Masked)
Transportation needs	Medical	79 041 (3.37)	1532 (8.9)	2302 (2.71)	43 328 (6.52)	22 231 (5.87)	496 (7.39)
	Non-medical	67 522 (2.87)	1348 (7.92)	1993 (2.37)	39 315 (5.82)	19 506 (5.24)	433 (6.75)
**Neighborhood-level SDoH**							
SVI	Socioeconomic status	1 456 906 (19.29)	23 798 (41.11)	69 526 (16.15)	1 066 925 (48.27)	744 119 (46.02)	6447 (19.42)
	Household characteristics	1 792 527 (23.73)	22 086 (38.15)	32 439 (7.54)	804 750 (36.41)	352 968 (21.83)	4286 (12.91)
	Racial and ethnic minority status	2 072 348 (27.43)	23 599 (40.77)	310 739 (72.19)	1 361 966 (61.62)	1 325 935 (82.01)	25 549 (76.96)
	Housing and transportation	2 277 128 (30.15)	30 540 (52.76)	159 343 (37.02)	1 087 879 (49.22)	879 485 (54.40)	15 604 (47.00)

Values expressed as N (%).

Abbreviations: AI/AN, American Indian or Alaskan Native; Black, Black or African American; Hispanic—Hispanic or Latino; IPV, interpersonal violence; NH/PI, Native Hawaiian or Other Pacific Islander; SVI, social vulnerability index.

### Individual-level SDoH

Below we describe completeness and burden for financial strain, food insecurity, housing instability, intimate partner violence, and transportation barriers.

1. ***Financial strain:*** After adjusting for age and gender, data completeness for financial resource strain varied by race/ethnicity, ranging from 18.1% (95% CI: 15.4%-21.0%) among Asian individuals to 22.8% (95% CI: 19.9%-26.0%) among White individuals (Table 3). Completeness was significantly lower for all non-White racial/ethnic groups compared to White individuals (Table 3). For example, the odds ratio (OR) for data completeness among Asian individuals compared to White individuals was 0.74 (95% CI: 0.67-0.82), indicating lower likelihood of documentation. Among individuals with available data, the prevalence of financial strain ranged from 30.8% among Asian individuals to 43.8% among Hispanic individuals (Table 4).
2. ***Food insecurity:*** The adjusted completeness of food insecurity (food scarcity and food worry) varied by data element and race/ethnicity, ranging from 24.8% for food scarcity among Asian individuals to 31.5% for food worry among White individuals. Adjusted data completeness was significantly lower for AI/AN, Asian, Hispanic, and NH/PI race/ethnicity groups compared to White individuals (Table 3). Among those with available data, the prevalence of food scarcity and food worry ranged from 4.2% and 5.0% among Asian individuals to 12.4% and 13.8% among Hispanic individuals, respectively (Table 4).
3. ***Housing instability:*** The adjusted completeness of homelessness and mortgage instability data also varied by data element and race/ethnicity, ranging from 15.7% for Asian individuals to 23.6% of White individuals (Table 3). As with food insecurity, adjusted data completeness for housing instability was significantly lower for AI/AN, Asian, Hispanic and NH/PI race/ethnicity groups compared to White individuals (Table 3). Among patients with available data, the prevalence of homelessness ranged from 1.3% in Asian individuals to 3.21% in Black individuals and 5.4% in AI/AN individuals, while mortgage instability ranged from 3.3% in Asians to 9.1% in Black individuals (Table 4).
4. ***Intimate partner violence (IPV):*** Data completeness for interpersonal violence also varied by data element and race/ethnicity, ranging from 11.2% for AI/AN to 15.6% for White individuals (Table 3). As with food insecurity and housing instability, adjusted data completeness for IPV was significantly lower for AI/AN, Asian, Hispanic, and NH/PI race/ethnicity groups compared to White individuals (Table 3). AI/AN reported higher levels of intimate partner violence compared to other groups (Table 4).
5. ***Transportation barriers:*** Medical and non-medical transportation needs were documented for 22.4% to 29.4% of individuals, depending on the attribute and race/ethnicity and followed similar patterns as other SDoH data elements (Table 3). AI/AN reported higher levels of unmet transportation needs compared to other groups (Table 4).

### Neighborhood-level SDoH

completeness of neighborhood-level SDoH (ie, SVI) are summarized in Table 3. Adjusted SVI completeness ranged from 94.7% for AI/AN to 99.3% for Asian individuals. Prevalence of socioeconomic vulnerability ranged from 16.1% for Asians to 48.3% for Black individuals. Housing and transportation vulnerabilities ranged from 30.1% for White to 54.4% for Hispanic individuals (Table 4).

## Discussion

### Summary of key findings

This study examined the completeness of SDoH data for individuals with T2D in the Epic Cosmos database. While individual-level SDoH variables—such as financial strain, food insecurity, housing instability, IPV, and transportation needs—provided granular, patient-specific insights, their documentation remained incomplete and inconsistent across racial and ethnic groups. Across all individual-level SDoH data elements and patient populations, adjusted completeness of data elements ranged between 11.2% for emotional IPV in AI/AN individuals to 31.5% for food worry in White individuals (Table 3).

### Progress in SDoH capture in recent years

Historically, documentation of SDoH in EHRs has been extremely limited. For example, in a national sample of 2013 hospital discharges, fewer than 2% of inpatient encounters had any documented ICD-9 SDoH codes (V codes).^22^ Similarly, a 2018 analysis of a health system’s database (∼5.4 million patients) found that structured EHR data recorded housing instability and financial strain for fewer than 1% of patients.^21^ Even in recent years, the adoption of ICD-10 SDoH codes (Z codes) remains low—one study across 12 US health systems found that the proportion of patients with any SDoH Z-code increased from 1.7% in 2016 to only 2.7% in 2021.^23^

Compared to these earlier estimates, our findings suggest that SDoH completeness has improved. The data still have significant gaps, but they now identify a substantial subset of patients with unmet social needs based on patients’ responses that differed from negative answers such as “Not hard at all,” “Never True,” and “No” (Table 1), offering opportunities for secondary analyses and targeted interventions. For example, AI/AN individuals experience higher transportation needs and may benefit from programs specifically designed to mitigate barriers in their communities.

### Remaining challenges

Although SDoH data completeness has improved in recent years, individual-level SDoH data remain insufficient for robust full-population secondary analyses. Additionally, we observed systematic under-collection of SDoH data among non-White patient populations, further exacerbating disparities in data availability. The incompleteness and differential capture of SDoH data not only limit the ability of clinical teams to effectively address social needs in patient care but also reduce the utility of these data for research and policy-driven interventions. It is important to note that Cosmos captures only the Epic-released SDoH screening questions. Organizations that collect SDoH information through customized workflows outside of Epic’s standard releases are not reflected in Cosmos. While there has been progress toward standardizing SDoH data capture, there is not yet a universally adopted standard for SDoH collection and exchange across all domains. These challenges highlight the urgent need for standardized, inclusive, and interoperable SDoH data collection practices to support both clinical decision-making and secondary analyses.

In addition to under-collection, misreporting of certain SDoH domains by patients, particularly those associated with stigma or potential legal implications, such as IPV, remains a significant challenge.^29^ Mandatory reporting laws for IPV-related injuries vary by state; in most states, providers are required to report injuries resulting from domestic violence to law enforcement.^30^ These reporting requirements may deter individuals from disclosing abuse due to fears of retaliation, loss of confidentiality, or involvement with the legal system. Estimates for physical IPV prevalence from the National Crime Victimization Survey (NCVS) data were 1.7 per 1000 in 2021 and 3.4 per 1000 in 2022,^31^ which are comparable to the EHR-based estimates in our dataset, which ranged from 1.8 to 9.9 per 1000 by racial/ethnic group (Table 4). Further research is needed to assess how stigma, legal context, and sociodemographic factors affect the accuracy and equity of IPV documentation in EHRs.

One critical concern with incomplete and biased SDoH documentation is its impact on AI model development. The increasing use of AI in T2D management presents both opportunities and challenges.^32^^,^^33^ AI models trained on incomplete or biased datasets may underrepresent vulnerable populations, leading to inequitable predictions and treatment recommendations.^34^^,^^35^ Missing SDoH variables can result in models that fail to adjust for social risks, disproportionately affecting racial and ethnic minority groups.^36^^,^^37^ Additionally, AI-driven risk stratification and clinical decision support tools may reinforce disparities if SDoH data are inconsistently captured across populations. Moreover, there may be differing selection biases within groups, not only between groups. Without complete social risk information, models may over-prioritize clinical factors while overlooking non-medical barriers to diabetes management, such as access to care, affordability of medications, or transportation challenges.^38^^,^^39^ As a result, socially disadvantaged patients may receive suboptimal treatment plans, further widening inequities in T2D outcomes. At the same time, the impacts of these apparent data biases on AI models are currently unknown; data biases do not automatically lead to biased models. Further research is necessary to determine the impact of these data biases on AI models.

### Potential solutions

To mitigate these challenges, EHR vendors and healthcare systems should standardize SDoH data collection through interoperable solutions. Significant progress has been made in this area through initiatives like the United States Core Data for Interoperability (USCDI)^40^ and the HL7 Da Vinci Gravity Project.^41^ USCDI has increasingly emphasized the inclusion of SDoH data in standardized EHR elements, with recent versions incorporating fields for food insecurity, housing status, and transportation needs. Meanwhile, the Gravity Project is actively developing HL7 Fast Healthcare Interoperability Resources (FHIR) implementation guides to facilitate structured capture and exchange of SDoH data across healthcare systems. These efforts aim to create a consistent framework for SDoH data sharing among payers, providers, and community-based organizations.

Beyond interoperability, stronger integration into routine clinical practice has to be achieved. Healthcare teams are already overburdened with documentation requirements, making it difficult to incorporate SDoH-related assessments into clinical workflows. Additionally, many providers hesitate to screen for SDoH due to a lack of resources to address identified social needs, further limiting data collection efforts. Developers of clinical decision support (CDS) interventions could consider interventions such as noninterruptive EHR alert to improve collection of SDoH.^42^ Additionally, EHR vendors could use large language models (LLMs) to extract SDoH from clinical notes to enrich structured EHR data and national EHR datasets.

To mitigate low completeness while EHR vendors, healthcare systems, and CDS developers work towards improving SDoH data capture, healthcare analysts and AI developers could adopt one of these 2 imperfect mitigation strategies: either use data augmentation strategies for missing data or use alternative variables such as neighborhood-level SDoH. The first approach involves bias mitigation strategies such as fairness-aware machine learning and model audits.^38^^,^^43^ Future research should explore data augmentation techniques to address missing SDoH information and evaluate the impact of SDoH-aware AI models on reducing health inequities.^44^^,^^45^ As a second approach, when appropriate, individual-level SDoH variables could be substituted with neighborhood-level and demographic variables. Neighborhood-level variables (eg, SVI) are available for almost all patients since addresses are routinely collected and mapped to external indices. However, such measures lack precision at the individual level and may not fully capture personal circumstances.^46^

### Limitations

This study has several limitations. First, it relies on retrospective EHR data, which are prone to documentation biases and missing information.^47^^,^^48^ For example, in some organizations, race and ethnicity data are collected based on patient self-report, while in others they are recorded by medical assistants without consulting the patients. While the Epic Cosmos database offers a comprehensive source of data from various healthcare environments, the generalizability of our findings may be constrained to individuals treated at institutions using the Epic EHR system. However, given that Epic Cosmos includes data from more than half of the US population, we believe our results are broadly applicable. Second, our study was limited to SDoH variables available in Epic Cosmos. Some essential SDoH variables are not currently collected in EHRs or are unavailable in the Epic Cosmos database. In addition, the variables available in Epic Cosmos provide only partial measures of the relevant SDoH. Third, we analyzed only structured SDoH data, while it is possible to extract additional SDoH information from clinical notes using natural language processing (NLP), LLMs, and other text mining methods.^49^ However, clinical notes were not available in Epic Cosmos at the time of analysis.

Fourth, SDoH data collection may vary across healthcare organizations, potentially affecting the validity of the findings. This limitation was addressed by using GEE to adjust for hierarchical nature of EHR data. Fifth, SDoH completeness could be influenced by the frequency of patient visits. Some individuals may have limited interactions with the healthcare system, which could impact whether their SDoH data were recorded.^50^ However, we note that SDoH data are expected to be collected and updated at each visit, including the first visit, according to best practices recommended by professional organizations.^51^ Sixth, this study was limited to data from the United States, which constrains generalizability to international settings. Future studies could explore how SDoH data are collected and structured in different countries to enhance cross-national comparisons and global applicability.

Seventh, this study focused specifically on patients with T2D. While we recognize the value of assessing SDoH completeness across the entire Epic Cosmos population, this broader analysis was beyond the available resources and objectives of this study. Expanding this research to other patient populations would provide further insights into SDoH documentation trends across different subpopulations. Eighth, we did not use the Surveillance, PREvention, and ManagEment of Diabetes Mellitus (SUPREME-DM) algorithm, which could have identified additional individuals with T2D.^52^ Instead, we focused on individuals with documented T2D diagnoses, using more restrictive inclusion criteria. Lastly, despite identifying a substantial number of patients with unmet social needs, this study could not compare the prevalence of unmet social needs across racial/ethnic groups due to systematic biases in data completeness.

## Conclusion

The study identifies concerning gaps in the completeness of individual-level SDoH variables. These findings underscore the need to recognize the value of SDoH in healthcare data systems and the importance of standardized data collection. However, the widespread availability of neighborhood-level SDoH variables demonstrates the potential for EHR data to support secondary analyses and equitable AI-model development as healthcare organizations work toward standardizing the collection of individual-level SDoH data.

## Data Availability

The data underlying this article cannot be shared publicly due to Epic Cosmos policies.

## References

[1] Shen Y , Yu J, Zhou J, Hu G. Twenty-five years of evolution and hurdles in electronic health records and interoperability in medical research: comprehensive review. J Med Internet Res. 2025;27:e59024. 10.2196/59024 39787599 PMC11757985

[2] Epic Cosmos. Accessed August 6, 2024. https://cosmos.epic.com/about/

[3] Palchuk MB , London JW, Perez-Rey D, et al A global federated real-world data and analytics platform for research. JAMIA Open. 2023;6:ooad035. 10.1093/JAMIAOPEN/OOAD035 37193038 PMC10182857

[4] The “All of Us” research program. New Engl J Med. 2019;381:668-676. 10.1056/NEJMSR1809937/SUPPL_FILE/NEJMSR1809937_DISCLOSURES.PDF 31412182 PMC8291101

[5] Haendel MA , Chute CG, Bennett TD; N3C Consortium, et al The National COVID Cohort Collaborative (N3C): rationale, design, infrastructure, and deployment. J Am Med Inform Assoc. 2021;28:427-443. 10.1093/JAMIA/OCAA196 32805036 PMC7454687

[6] Forrest CB , McTigue KM, Hernandez AF, et al PCORnet^®^ 2020: current state, accomplishments, and future directions. J Clin Epidemiol. 2021;129:60-67. 10.1016/J.JCLINEPI.2020.09.036 33002635 PMC7521354

[7] Castellanos EH , Wittmershaus BK, Chandwani S. Raising the bar for real-world data in oncology: approaches to quality across multiple dimensions. JCO Clin Cancer Inform. 2024;8:e2300046. 10.1200/CCI.23.00046 38241599 PMC10807898

[8] NEJM Catalyst. Social determinants of health (SDOH). Published online 2017. Accessed March 8, 2025. 10.1056/CAT.17.0312

[9] World Health Organization: Social Determinants of Health. Accessed March 8, 2025. https://www.who.int/health-topics/social-determinants-of-health#tab=tab_1

[10] Hill-Briggs F , Adler NE, Berkowitz SA, et al Social determinants of health and diabetes: a scientific review. Diabetes Care. 2020;44:258-279. 10.2337/dci20-0053 33139407 PMC7783927

[11] Hill-Briggs F , Ephraim PL, Vrany EA, et al Social determinants of health, race, and diabetes population health improvement: Black/African Americans as a population exemplar. Curr Diab Rep. 2022;22:117-128. 10.1007/s11892-022-01454-3 35239086 PMC8891426

[12] Hill-Briggs F , Fitzpatrick SL. Overview of social determinants of health in the development of diabetes. Diabetes Care. 2023;46:1590-1598. 10.2337/dci23-0001 37354331

[13] Centers for Disease Control and Prevention. *National Diabetes Statistics Report*. Centers for Disease Control and Prevention; 2023.

[14] Lin J , Thompson TJ, Cheng YJ, et al Projection of the future diabetes burden in the United States through 2060. Popul Health Metr. 2018;16:9. 10.1186/S12963-018-0166-4 29903012 PMC6003101

[15] CMS Strategic Plan Health EquityPublished online 2024. Accessed August 14, 2025. https://www.cms.gov/files/document/health-equity-fact-sheet.pdf

[16] Dullabh P , Hovey L, Leaphart D, Chiao A, Heaney-Huls K. Expanding Social Determinants of Health Data across PCORnet^®^ Clinical Research Networks. Patient Centered Outcomes Research Institute; 2022. White paper. Prepared by NORC at the University of Chicago under Contract No. IDIQ-TO#27-NORC-SCI-AOSEPP-05-24-2021.

[17] TechTarget. Epic’s EHR optimization mitigates SDOH, promotes care coordination. Accessed August 1, 2024. https://www.techtarget.com/searchhealthit/news/366579171/Epics-EHR-Optimization-Mitigates-SDOH-Promotes-Care-Coordination

[18] Craven CK , Highfield L, Basit M, et al Toward standardization, harmonization, and integration of social determinants of health data: a Texas Clinical and Translational Science Award institutions collaboration. J Clin Transl Sci. 2024;8:e17. 10.1017/cts.2024.2 38384919 PMC10880009

[19] Phuong J , Zampino E, Dobbins N, et al Extracting patient-level social determinants of health into the OMOP common data model. AMIA Annu Symp Proc. 2021;2021:989-998. 35308947 PMC8861735

[20] Cook LA , Sachs J, Weiskopf NG. The quality of social determinants data in the electronic health record: a systematic review. J Am Med Inform Assoc. 2021;29:187-196. 10.1093/JAMIA/OCAB199 34664641 PMC8714289

[21] Hatef E , Rouhizadeh M, Tia I, et al Assessing the availability of data on social and behavioral determinants in structured and unstructured electronic health records: a retrospective analysis of a multilevel health care system. JMIR Med Inform. 2019;7:e13802. 10.2196/13802 31376277 PMC6696855

[22] Torres JM , Lawlor J, Colvin JD, et al ICD social codes: an underutilized resource for tracking social needs. Med Care. 2017;55:810-816. 10.1097/MLR.0000000000000764 28671930

[23] Llamocca EN , Ahmedani BK, Lockhart E, et al Use of ICD-10-CM codes for adverse social determinants of health across health systems. Psychiatr Serv. 2025;76:22-29. 10.1176/APPI.PS.20240148 39308169

[24] Tarabichi Y , Frees A, Honeywell S, et al The Cosmos collaborative: a vendor-facilitated electronic health record data aggregation platform. ACI Open. 2021;5:e36-e46. 10.1055/S-0041-1731004 35071993 PMC8775787

[25] Cheng YJ , Kanaya AM, Araneta MRG, et al Prevalence of diabetes by race and ethnicity in the United States, 2011-2016. JAMA. 2019;322:2389-2398. 10.1001/JAMA.2019.19365 31860047 PMC6990660

[26] Benchimol EI , Smeeth L, Guttmann A, RECORD Working Committee, et al The REporting of studies Conducted using Observational Routinely-collected health Data (RECORD) statement. PLoS Med. 2015;12:e1001885. 10.1371/JOURNAL.PMED.1001885, 26440803 PMC4595218

[27] CDC/ATSDR Social Vulnerability Index (SVI). Accessed August 14, 2025. https://www.atsdr.cdc.gov/place-health/php/svi/index.html

[28] Office of Management and Budget. Revisions to OMB’s Statistical Policy Directive No. 15: Standards for Maintaining, Collecting, and Presenting Federal Data on Race and Ethnicity. 2024. Accessed August 14, 2025. https://spd15revision.gov/content/spd15revision/en/2024-spd15/categories-definitions.html

[29] Overstreet NM , Quinn DM. The intimate partner violence stigmatization model and barriers to Help-Seeking. Basic Appl Soc Psych. 2013;35:109-122. 10.1080/01973533.2012.746599 23524454 PMC3601798

[30] Which states have mandatory domestic violence reporting? Accessed May 12, 2025. https://mandatedreportertraining.com/resources/blog/which-states-have-mandatory-domestic-violence-reporting/? utm_source=chatgpt.com

[31] Thompson A , Tapp SN, Statisticians B. Criminal victimization, 2022. Published online 2023. Accessed May 12, 2025. https://ncvs.bjs.ojp.gov

[32] Fujihara K , Sone H. Machine learning approach to drug treatment strategy for diabetes care. Diabetes Metab J. 2023;47:325-332. 10.4093/DMJ.2022.0349 36631990 PMC10244197

[33] Sheng B , Pushpanathan K, Guan Z, et al Artificial intelligence for diabetes care: current and future prospects. Lancet Diabetes Endocrinol. 2024;12:569-595. 10.1016/S2213-8587(24)00154-2 39054035

[34] Obermeyer Z , Powers B, Vogeli C, Mullainathan S. Dissecting racial bias in an algorithm used to manage the health of populations. Science. 2019;366:447-453. 10.1126/science.aax2342 31649194

[35] Rajkomar A , Hardt M, Howell MD, Corrado G, Chin MH. Ensuring fairness in machine learning to advance health equity. Ann Intern Med. 2018;169:866-872. 10.7326/M18-1990 30508424 PMC6594166

[36] Gianfrancesco MA , Tamang S, Yazdany J, Schmajuk G. Potential biases in machine learning algorithms using electronic health record data. JAMA Intern Med. 2018;178:1544-1547. 10.1001/JAMAINTERNMED.2018.3763 30128552 PMC6347576

[37] Challen R , Denny J, Pitt M, Gompels L, Edwards T, Tsaneva-Atanasova K. Artificial intelligence, bias and clinical safety. BMJ Qual Saf. 2019;28:231-237. 10.1136/BMJQS-2018-008370 PMC6560460 30636200

[38] Chin MH , Afsar-Manesh N, Bierman AS, et al Guiding principles to address the impact of algorithm bias on racial and ethnic disparities in health and health care. JAMA Netw Open. 2023;6:E2345050. 10.1001/JAMANETWORKOPEN.2023.45050 38100101 PMC11181958

[39] Dorr DA , Adams L, Embí P. Harnessing the promise of artificial intelligence responsibly. JAMA. 2023;329:1347-1348. 10.1001/JAMA.2023.2771 36972068

[40] United States Core Data for Interoperability (USCDI). Interoperability Standards Platform (ISP). Accessed February 26, 2025. https://www.healthit.gov/isp/united-states-core-data-interoperability-uscdi

[41] Gravity project. Accessed February 26, 2025. https://thegravityproject.net/

[42] Lenert L , Rheingold AA, Simpson KN, et al Electronic health record-based screening for intimate partner violence: a cluster randomized clinical trial. JAMA Netw Open. 2024;7:e2425070. 10.1001/JAMANETWORKOPEN.2024.25070, 39088215 PMC11294960

[43] Chen IY , Pierson E, Rose S, Joshi S, Ferryman K, Ghassemi M. Ethical machine learning in healthcare. Annu Rev Biomed Data Sci. 2021;4:123-144. 10.1146/ANNUREV-BIODATASCI-092820-114757 34396058 PMC8362902

[44] Madley-Dowd P , Hughes R, Tilling K, Heron J. The proportion of missing data should not be used to guide decisions on multiple imputation. J Clin Epidemiol. 2019;110:63-73. 10.1016/J.JCLINEPI.2019.02.016 30878639 PMC6547017

[45] Pedersen AB , Mikkelsen EM, Cronin-Fenton D, et al Missing data and multiple imputation in clinical epidemiological research. Clin Epidemiol. 2017;9:157-166. 10.2147/CLEP.S129785 28352203 PMC5358992

[46] Brown EM , Franklin SM, Ryan JL, et al Assessing area-level deprivation as a proxy for Individual-Level social risks. Am J Prev Med. 2023;65:1163-1171. 10.1016/J.AMEPRE.2023.06.006 37302512

[47] Al-Sahab B , Leviton A, Loddenkemper T, Paneth N, Zhang B. Biases in electronic health records data for generating real-world evidence: an overview. J Healthc Inform Res. 2024;8:121-139. 10.1007/S41666-023-00153-2 38273982 PMC10805748

[48] Verheij RA , Curcin V, Delaney BC, McGilchrist MM. Possible sources of bias in primary care electronic health record data use and reuse. J Med Internet Res. 2018;20:e185. 10.2196/JMIR.9134 29844010 PMC5997930

[49] Brown JR , Ricket IM, Reeves RM, et al Information extraction from electronic health records to predict readmission following acute myocardial infarction: does natural language processing using clinical notes improve prediction of readmission? J Am Heart Assoc. 2022;11:e024198. 10.1161/JAHA.121.024198 35322668 PMC9075435

[50] Weber GM , Adams WG, Bernstam EV, et al Biases introduced by filtering electronic health records for patients with “complete data.” J Am Med Inform Assoc. 2017;24:1134-1141. 10.1093/JAMIA/OCX071 29016972 PMC6080680

[51] Magoon V. Screening for social determinants of health in daily practice. Fam Pract Manag. 2022;29:6-11. Accessed March 6, 2025. https://www.aafp.org/pubs/fpm/issues/2022/0300/p6.html 35290006

[52] Nichols GA , Desai J, Lafata JE, et al; SUPREME-DM Study Group. Construction of a multisite DataLink using electronic health records for the identification, surveillance, prevention, and management of diabetes mellitus: the SUPREME-DM project. Prev Chronic Dis. 2012;9:E110. 10.5888/PCD9.110311 22677160 PMC3457753

